# Integrating Precision Sepsis Risk Stratification Into Systems-Based Antimicrobial Stewardship

**DOI:** 10.1093/ofid/ofag162

**Published:** 2026-03-21

**Authors:** James S Ford, Shamim Nemati, Ben Gross, Atul Malhotra, Gabriel Wardi

**Affiliations:** Department of Emergency Medicine, University of California San Diego, La Jolla, California, USA; Division of Biomedical Informatics, University of California, San Diego, La Jolla, California, USA; Department of Medicine, University of California San Diego, San Diego, La Jolla, California, USA; Division of Biomedical Informatics, University of California, San Diego, La Jolla, California, USA; Department of Medicine, University of California San Diego, San Diego, La Jolla, California, USA; Department of Emergency Medicine, University of California San Diego, La Jolla, California, USA; Department of Medicine, University of California San Diego, San Diego, La Jolla, California, USA

**Keywords:** antibiotics, antimicrobial stewardship, machine learning, sepsis

## Abstract

Early broad-spectrum antimicrobial administration in sepsis is strongly associated with improved survival. However, widespread implementation of time-based antibiotic mandates has substantially increased antimicrobial utilization, contributing to escalating global antimicrobial resistance. This creates a fundamental tension in sepsis care: Aggressive empiric therapy optimizes short-term outcomes for individual patients but threatens long-term antimicrobial effectiveness at a population level. This perspective frames the “sepsis–antimicrobial stewardship tension” as a systems-level problem driven by diagnostic uncertainty, organizational incentives, and guideline implementation. We review emerging evidence demonstrating heterogeneity in the benefit of early antibiotics and highlight how rigid quality metrics, such as the US Centers for Medicare & Medicaid Services SEP-1 measure, may exacerbate unnecessary antimicrobial use. Finally, we discuss how advances in artificial intelligence, machine learning, and host–response diagnostics can enable precision risk-guided antimicrobial timing. Integrating these tools into clinical workflows via health systems engineering offers a path toward addressing antimicrobial resistance.

Sepsis, a dysregulated host response to infection, has a remarkably high mortality when not identified and treated early. In 2004, the Surviving Sepsis Campaign released the first formalized international guideline to recommend prompt administration of broad-spectrum antimicrobials in patients with suspected sepsis, and over the next decade seminal literature emerged demonstrating a clear time-dependent mortality benefit with timely administration [1, 2]. Since then, many other national and international guidelines have adopted similar recommendations, solidifying this practice into standard of care [3, 4].

While these guidelines have been instrumental in standardizing best practices and saving lives, increased broad-spectrum antimicrobial (BSA) use has unequivocably contributed to rising rates of antimicrobial resistance (AMR). Globally, antibiotic consumption increased approximately 65% between 2000 and 2015, and another 16% between 2016 and 2023 [5]. The downstream effect on AMR is harrowing: A recent report from the World Health Organization (WHO) indicates that in high-AMR regions, 1 in 3 bacterial infections are resistant to treatment, and it is estimated that AMR contributes to the death of nearly 5 million people every year [6]. This escalating global crisis underscores that the current approach to sepsis management is no longer sustainable at a population level. Electing the most beneficent antimicrobial policy requires considering a complex tradeoff: Aggressive early broad-spectrum therapy maximizes short-term patient outcomes but accelerates long-term resistance, while more judicious use protects future antimicrobial efficacy but carries a short-term risk of delayed treatment in patients with diagnostic uncertainty.

This “sepsis–antimicrobial stewardship tension” has been described as a systems-level challenge that is affected by “organizational pressures, inadequate diagnostic tools, and sociocultural factors” [7]. Lounsbury et al. describe how this tension can be viewed through the lens of the Systems Engineering Initiative for Patient Safety (SEIPS) framework, which emphasizes that patient outcomes are affected not just by individual performance but by how individual components of the health system are designed and interact. This framing is particularly important to the management of sepsis, where the practice of delivering early antibiotics, which has long been associated with improved survival, has become institutionalized through national and international guidelines. The Surviving Sepsis Campaign's (SSC’s) Sepsis-3 Guidelines provide antibiotic timing (eg, ≤1 hour, ≤3 hours) recommendations according to sepsis severity and diagnostic certainty; however, there is growing evidence that early antibiotic administration (ie, <1 hour) is necessary only in patients with septic shock, and antibiotics can be safely delayed in lower-risk patients, highlighting the opportunity for improved antimicrobial stewardship [8]. These findings challenge the implicit assumption that all patients meeting sepsis criteria derive uniform benefit from immediate antimicrobial administration.

In the United States, the Centers for Medicare and Medicaid (CMS) Severe Sepsis and Septic Shock Management bundle (SEP-1) mandates antibiotics within 3 hours for all patients meeting severe sepsis criteria (Sepsis-2) [4]. However, given the low specificity of using the systemic inflammatory response syndrome (SIRS) construct as part of its operational definition of sepsis, it is challenging to differentiate infected from noninfected patients within this time frame, making it unsurprising that SEP-1 implementation has been associated with increased broad-spectrum antibiotic utilization, without clear mortality benefit [9]. Importantly, SEP-1 was recently adopted into the CMS Hospital Value Based Purchasing Program, a program that withholds 2% of hospitals’ inpatient Medicare payments and redistributes those funds based on each hospital's performance in terms of quality, safety, patient experience, and efficiency measures. This federal policy, now tied to a large fiscal incentive, will likely increase pressure on administrators and frontline providers to comply with the antibiotic mandate, and likely lead to increased antimicrobial use in patients who may or may not have sepsis. As Lounsbury et al. highlight, these “organizational pressures” expose the gap between “work-as-imagined” and “work-as-done” and illustrate why system redesign is needed to improve antimicrobial stewardship. In short, clinicians need an objective, data-driven mechanism to balance urgency against diagnostic uncertainty when evaluating patients for possible sepsis.

Emerging technologies in artificial intelligence (AI) and high-resolution omics offer new avenues that could transform sepsis diagnostics, prognostics, and therapeutics in the coming decade. For example, real-time sepsis risk stratification offers a promising path toward systems-level change. In the United Kingdom, the National Institute for Health and Care Excellence (NICE) recommends use of the National Early Warning Score 2 (NEWS2) to help inform antimicrobial timing based on illness severity [10]. Building on this principle, machine learning methods can augment traditional scoring systems by continuously integrating real-time clinical data to generate personalized risk predictions. Indeed, in a recent multicenter study, 2 deep learning models were used to objectively stratify patients into SSC risk groups according to risk of sepsis and risk of developing shock [11]. This study demonstrated that antibiotic administration was strongly associated with survival among the highest-risk patients (“sepsis probable,” regardless of shock risk), whereas modest delays were not linked to excess mortality in low-risk groups (“sepsis possible,” low risk of developing shock) [11]. In another study by our group, we demonstrated that large language models could be used to improve a deep learning sepsis risk prediction system and reduce false positives in patients with high diagnostic uncertainty [12]. Importantly, models must be carefully integrated into care pathways to ensure that alert triggers arrive *before* sepsis has been identified and treatment decisions have been made, a timing and workflow alignment challenge reported in several real-world evaluations of sepsis early warning systems [13, 14]. A recent study by Liesenfeld et al. described a new testing platform that combines transcriptomics and machine learning to determine the likelihood of bacterial vs viral infection and the need for critical care interventions within 7 days, with higher accuracy than conventional tests such as C-reactive protein, procalcitonin, or white blood cell count [15]. Together, these tools offer a way to objectively quantify risk and augment clinical gestalt.

The integration of these emerging sepsis risk prediction systems into clinical workflows can help clinicians balance competing priorities by objectively quantifying risk, providing a shared mental model for the treatment team, and establishing clear treatment recommendations according to risk. For example, when algorithms identify low-risk patients, the system itself can document and justify a watchful waiting approach, aligning antimicrobial stewardship with patient safety and organizational accountability. Furthermore, risk prediction systems can be implemented alongside structured feedback systems for antimicrobial prescribing—together, these systems can augment clinician behavior and institutional culture over time. At the institutional level, integrating precision risk models with treatment recommendations provides a practical mechanism for operationalizing systems-based engineering into evidence-based clinical practice. While AI-based tools have shown promise, they also have limitations that must be appropriately addressed. For example, models may have high false-positive rates, can have system-level biases if not externally validated, and can be susceptible to treatment-outcome loops that may distort causal relationships and bias future predictions. Hence, thoughtful, prospective implementation of AI-informed tools into precision care pathways is needed to fully elucidate their real-world impact on antimicrobial stewardship practices and patient outcomes. Lastly, we recommend that federal quality programs and guideline committees recalibrate sepsis mandates to allow for risk-stratified flexibility in antibiotic timing, supported by prospective validation studies, rather than continuing to enforce uniform time-based thresholds that may not reflect underlying patient risk, and that inadvertently lead to antimicrobial overuse.
